# Phorbol ester reduces ethanol excitation of dopaminergic neurons of the ventral tegmental area: involvement of protein kinase C theta

**DOI:** 10.3389/fnint.2013.00096

**Published:** 2013-12-25

**Authors:** Sudarat Nimitvilai, Devinder S. Arora, Chang You, Maureen McElvain, Mark S. Brodie

**Affiliations:** ^1^Department of Neurosciences, Medical University of South CarolinaCharleston, SC, USA; ^2^School of Pharmacy, Griffith UniversityGold Coast Campus, QLD, Australia; ^3^Department of Physiology and Biophysics, University of Illinois at ChicagoChicago, IL, USA

**Keywords:** PKC theta, ethanol, protein kinase C, electrophysiology, brain slices, alcohol, reward, dopamine

## Abstract

Neurons of the ventral tegmental area (VTA) play a key role in the rewarding and reinforcing effects of drugs of abuse, including alcohol. Ethanol directly increases the firing rate of dopaminergic (DAergic) VTA neurons, but modulation of the firing rate of DAergic VTA neurons can be controlled by a number of factors, including some that are under the control of protein kinase C (PKC). Application of phorbol esters activates PKC and the present study assessed the effect of a phorbol ester, phorbol 12-myristate 13-acetate (PMA), on ethanol-induced excitation of DA VTA neurons. Ethanol-induced excitation of DAergic VTA neurons was reduced significantly in the presence of PMA. This action of PMA was antagonized by chelerythrine chloride, a non-selective antagonist of PKC, but not by moderate concentrations of antagonists of conventional PKC isoforms (Gö6976 and Gö6983). A PKC δ/θ inhibitor antagonized PMA-induced reduction of ethanol excitation. Since PKCδ antagonist Gö6983 did not antagonize the effect of PMA on ethanol excitation, the PMA reduction of ethanol excitation is most likely to be mediated by PKCθ. Antagonists of intracellular calcium pathways were ineffective in antagonizing PMA action on ethanol excitation, consistent with the lack of calcium dependence of PKCθ. In summary, ethanol-induced excitation of VTA neurons is attenuated in the presence of PMA, and this attenuation appears to be mediated by PKCθ. This novel mechanism for interfering with ethanol activation of reward-related neurons could provide a new target for pharmacotherapy to ameliorate alcoholism.

## INTRODUCTION

Dopaminergic (DAergic) neurons of the ventral tegmental area (VTA) are involved in the mediation of rewarding and reinforcing properties of numerous stimuli, including abused drugs such as ethanol and cocaine (Wise, 1996; Di Chiara and Imperato, 1988). DAergic VTA neurons produce action potentials spontaneously both *in vivo* (Bunney et al., 1973) and *in vitro* (Brodie and Dunwiddie, 1987), and are regulated by dopamine D2 autoreceptors that inhibit the firing of these neurons (Grace, 1987). In addition, the firing rates of DAergic neurons of the VTA can be modulated by a number of neurotransmitters such as glutamate, GABA, and neurotensin (Kalivas, 1993; Adell and Artigas, 2004).

Ethanol produces numerous specific actions on DAergic neurons in the VTA. For example, acute ethanol increases h-current (Brodie and Appel, 1998), reduces M-current (Koyama et al., 2007), and increases barium-sensitive potassium current (McDaid et al., 2008). In addition, ethanol enhances glutamatergic (Deng et al., 2009) and GABAergic post-synaptic potentials (Theile et al., 2008). Some actions of ethanol may directly cause the phenotypic response to ethanol (e.g., increased firing Gessa et al., 1985; Brodie et al., 1990) and other effects may not directly play a role (McDaid et al., 2008), but may be involved in modulating those direct effects. We have reported the phenomenon of dopamine inhibition reversal (DIR): long-duration administration of moderate concentrations of dopamine results in a time-dependent decrease in dopamine-induced inhibition. DIR requires concurrent stimulation of D2 and D1/D5 dopamine receptors (Nimitvilai and Brodie, 2010). In addition, we found that low concentrations of ethanol (10–80 mM) block the reversal of quinpirole inhibition produced by PMA (Nimitvilai et al., 2012b), indicating that ethanol interferes with DIR at a step at or subsequent to the activation of PKC. Therefore, within the VTA, there are effects of ethanol on physiological processes.

The action of ethanol on PKC has been reported to regulate the functions of numerous receptors and cell activities (Stubbs and Slater, 1999; for review see Newton and Messing, 2006). Responses to both GABA and glutamate are altered when PKC is activated; GABA_A_ responses are enhanced through activation of PKC (Wafford and Whiting, 1992; Weiner et al., 1994; Harris et al., 1995), and AMPA/kainate receptors are inhibited by PKC (Dildy-Mayfield and Harris, 1995). Despite these and other findings, the role of PKC in ethanol action in the VTA is not clear.

The primary effect of ethanol on DAergic VTA neurons is to increase the firing rate (Brodie et al., 1990) but a careful assessment of the effects of PKC activation on firing of DAergic VTA neurons has not been performed. Our previous study showed that DIR (during which there is activation of conventional PKCs) does not result in a reduction of ethanol excitation, but direct activation of protein kinase C by PMA does cause a reduction of sensitivity to ethanol excitation (Nimitvilai et al., 2012b). In the present study, therefore, we extended these observations to determine the mechanism underlying the effect of PMA on ethanol-induced excitation.

## MATERIALS AND METHODS

### ANIMALS

Male Fischer 344 (F344; 90–150 gm) rats used in these studies were obtained from Harlan Laboratories, Inc. (Indianapolis, IN, USA). All rats were treated in strict accordance with the NIH Guide for the Care and Use of Laboratory Animals and all experimental methods were approved by the Animal Care Committee of the University of Illinois at Chicago.

### PREPARATION OF BRAIN SLICES

Brain slices containing the VTA were prepared from the subject animals as previously described (Brodie et al., 1999a). Briefly, following rapid removal of the brain, the tissue was blocked coronally to contain the VTA and substantia nigra; the cerebral cortices and a portion of the dorsal mesencephalon were removed. The tissue block was mounted in the vibratome and submerged in chilled cutting solution. The composition of the cutting solution was (in mM): KCl 2.5, CaCl_2_ 2.4, MgSO_4_ 1.3, NaHCO_3_ 26, glucose 11, and sucrose 220. Both solutions were saturated with 95% O_2_/ 5% CO_2_ (pH = 7.4). Coronal sections (400 μm thick) were cut and the slice was placed onto a mesh platform in the recording chamber. The slice was totally submerged in aCSF maintained at a flow rate of 2 ml/min; the temperature in the recording chamber was kept at 35°C. The composition of the aCSF in these experiments was (in mM): NaCl 126, KCl 2.5, NaH_2_PO_4_ 1.24, CaCl_2_ 2.4, MgSO_4_ 1.3, NaHCO_3_ 26, glucose 11. Equilibration time of at least one hour was allowed after placement of tissue in the recording chamber before electrodes were placed in the tissue.

### CELL IDENTIFICATION

The VTA was clearly visible in the fresh tissue as a grey area medial to the darker substantia nigra, and separated from the nigra by white matter. Recording electrodes were placed in the VTA under visual control. Putative DA dopaminergic neurons (DAergic neurons) have been shown to have distinctive electrophysiological characteristics (Grace and Bunney, 1984; Lacey et al., 1989). Only those neurons which were anatomically located within the VTA and which conformed to the criteria for DAergic neurons established in the literature and in this laboratory (Lacey et al., 1989; Mueller and Brodie, 1989) were studied. These criteria include broad action potentials (2.5 ms or greater, measured as the width of the bi- or tri-phasic waveform at the baseline), slow spontaneous firing rate (0.5–5 Hz), and a regular interspike interval. Cells were not tested with opiate agonists as has been done by other groups to further characterize and categorize VTA neurons (Margolis et al., 2006). Some neurons with the characteristics we used to identify DA VTA neurons may not, in fact, be DA-containing (Margolis et al., 2006).

### DRUG ADMINISTRATION

Drugs were added to the aCSF by means of a calibrated infusion pump from stock solutions 100–1000 times the desired final concentrations. The addition of drug solutions to the aCSF was performed in such a way as to permit the drug solution to mix completely with aCSF before this mixture reached the recording chamber. Final concentrations were calculated from aCSF flow rate, pump infusion rate and concentration of drug stock solution. The small volume chamber (about 300 μl) used in these studies permitted the rapid application and washout of drug solutions. Typically drugs reach equilibrium in the tissue after 2–3 min of application.

In some experiments, drugs were added to the extracellular microelectrode filling solution (0.9% NaCl) at a concentration 10 times greater than that which would have been used in the extracellular medium. To allow time for the drug to diffuse from the pipette to the cell, the effects of pipette-applied drugs were tested no less than 20 min after initiating the recording. This allowed time for the drug to diffuse from the pipette and reach the cell of interest. Although we have no way of measuring the concentration of the drug after dilution during the diffusion, we have obtained similar results when we have compared the effects of a given drug with a pipette concentration 10-fold higher than a bath concentration (data not shown). Pipette delivery has the advantage of more localized drug application and reduced expense. Such local delivery of drugs through recording pipettes has been used in the past by our lab and others (Pesavento et al., 2000; Nimitvilai et al., 2012a; Nimitvilai et al., 2013).

Ethanol, dopamine, and most of the salts used to prepare the extracellular media were purchased from Sigma (St. Louis, MO, USA). Phorbol 12-myristate 13-acetate (PMA), chelerythrine chloride, Gö6976 (5,6,7,13-tetrahydro-13-methyl-5-oxo-12H-indolo[2,3-a]pyrrolo[3,4c]carbazole-12-propanenitrile), Gö6983 (3-[1-[3-(Dimethylamino)propyl]-5-methoxy-1 H-indol-3-yl]-4-(1H-indol-3-yl)-1H-pyrrole-2,5-dione), ryanodine, 2-aminoe-thoxydiphenyl borate (2-APB), and dantrolene were purchased from Tocris (Minneapolis, MN). PKCδ/θ inhibitor (5-(3,4-Dimeth-oxyphenyl)-4-(1H-indol-5-ylamino)-3-pyridinecarboni trile) was purchased from Calbiochem^®^ (Billercia, MA, USA).

### EXTRACELLULAR RECORDING

All recordings used an extracellular recording technique, which was chosen for these studies as this method permits the recordings to be stable and of long duration (routinely >1 h) and allows us to assess the effects of extended exposure (>30 min) to drugs. The limitation of only measuring spontaneous action potential frequency (rather than membrane potential or other electro-physiological parameters) is counterbalanced by the advantage of being able to determine the time course of drug actions and interactions. Extracellular recording electrodes were made from 1.5 mm diameter glass tubing with filament and were filled with 0.9% NaCl. Tip resistance of the microelectrodes ranged from 2 to 4 MΩ. An extracellular amplifier was used in conjunction with an IBM-PC-based data acquisition system (ADInstruments, Inc,). Offline analysis was used to calculate, display and store the frequency of firing over 1-min intervals. Additional software was used to calculate the firing rate over 5-s intervals. Firing rate was determined before and during drug application. Firing rate was calculated over 1 min intervals prior to administration of drugs and during the drug effect; peak drug-induced changes in firing rate were expressed as the percentage change from the control firing rate according to the formula (FRD – FRC/FRC) × 100, where FRD is the firing rate during the peak drug effect and FRC is the control firing rate. The change in firing rate thus is expressed as a percentage of the initial firing rate, which controls for small changes in firing rate which may occur over time. This formula was used to calculate both excitatory and inhibitory drug effects. Peak excitation was defined as the peak increase in firing rate produced by the drug (e.g., ethanol) greater than the pre-drug baseline. Inhibition was defined as the lowest firing rate below the pre-drug baseline. Inhibition reversal was observed as a statistically significant reduction in the inhibition.

### DATA COLLECTION

For comparison of the time course of effects on firing rate, the data were normalized and averaged. Firing rates over 1 min intervals were calculated and normalized to the 1-min interval immediately prior to the DA administration. These normalized data were averaged by synchronizing the data to the drug administration period, and graphs of the averaged data were made.

### STATISTICAL ANALYSIS

Averaged numerical values were expressed as the mean ± the standard error of the mean (SEM). The differences among firing rates were assessed with one-way repeated measures ANOVA, followed by Tukey *post hoc* comparisons (Kenakin, 1987). Statistical analyses were performed with Origin 8.5 (Originlab Corporation, Northampton, MA, USA).

## RESULTS

### VTA NEURON CHARACTERISTICS

A total of 172 VTA neurons were examined. All neurons in normal extracellular medium had regular firing rates and ranged from 0.72 to 4.58 Hz, with a mean of 2.13 ± 0.07 Hz. In the course of performing the experiments described below, we used a number of pharmacological agents, delivered either via the extracellular medium or via the recording pipette, and these agents were applied for 20 min before the administration of ethanol. The effects of these chemicals alone on changes in firing rate of DAergic VTA neurons are shown in **Table 1**; the mean firing rates shown in the table are the pre-drug baseline, the firing rate at 20 min time point before ethanol administration, and the percentage change in firing rate at 20 min time point compared to the pre-drug baseline. Note that there was no significant change in firing rate induced by most of the treatments (12 of 18) and in only two cases was there a change greater than 10%. Cells which did not return to at least 65% of their pre-drug firing rate during this washout were not used. One benefit of the extracellular recording method used in these studies is that long duration recordings can be made reliably; the average recording duration was 76.86 ± 1.26 min, with a range of 74–170 min.

**Table 1:** Changes in firing rate in response to PMA and/or inhibitors.

Chemical name	Chemical conc. (μM)	Number of cells	Mean firing rate at baseline (Hz)	Mean firing rate at 20 min (Hz)	Change in firing rate (%)	*p*-value
1%DMSO	–	7	1.68 ± 0.23	1.66 ± 0.21	-0.5 ± 0.7	>0.05
PMA	1	17	1.9 ± 0.16	1.78 ± 0.17	-6.7 ± 2.4	<0.05
Gö6976 (+PMA)	10	8	1.52 ± 0.11	1.46 ± 0.12	-3.8 ± 1.9	>0.05
Gö6976 (+1%DMSO)	10	9	1.95 ± 0.19	1.89 ± 0.13	-0.53 ± 4.72	>0.05
Chelerythrine (+PMA)	10	15	2.42 ± 0.26	2.21 ± 0.22	-6.70 ± 3.0	<0.05
Chelerythrine (+1%DMSO)	10	7	1.78 ± 0.38	1.68 ± 0.31	-4.70 ± 3.9	>0.05
Gö6983 (+PMA)	10	12	2.54 ± 0.30	2.39 ± 0.27	-5.1 ± 2.19	<0.05
Gö6983 (+1%DMSO)	10	5	3.47 ± 0.42	3.35 ± 0.43	-4.09 ± 1.85	>0.05
PKCδ/θ (+PMA)	0.7	12	2.02 ± 0.21	1.90 ± 0.22	-6.72 ± 3.15	<0.05
PKCδ/θ (+1%DMSO)	0.7	11	2.81 ± 0.29	2.78 ± 0.28	-0.94 ± 2.41	>0.05
2-APB (+PMA)	10	8	2.12 ± 0.19	1.77 ± 0.18	-16.77 ± 2.1	<0.05
2-APB (+1%DMSO)	10	10	1.84 ± 0.19	1.67 ± 0.14	-6.9 ± 3.2	>0.05
Ryanodine (+PMA)	10	8	2.18 ± 0.21	1.93 ± 0.17	-10.3 ± 3.7	<0.05
Ryanodine (+1%DMSO)	10	7	2.25 ± 0.37	2.09 ± 0.28	-4.8 ± 4.1	>0.05
Dantrolene (+PMA)	20	10	1.85 ± 0.18	1.90 ± 0.11	9.27 ± 10.0	>0.05
Dantrolene (+1%DMSO)	20	5	1.47 ± 0.27	1.46 ± 0.28	-0.38 ± 4.09	>0.05

### ETHANOL EXCITATION IS REDUCED IN THE PRESENCE OF PMA

**FIGURE 1:**
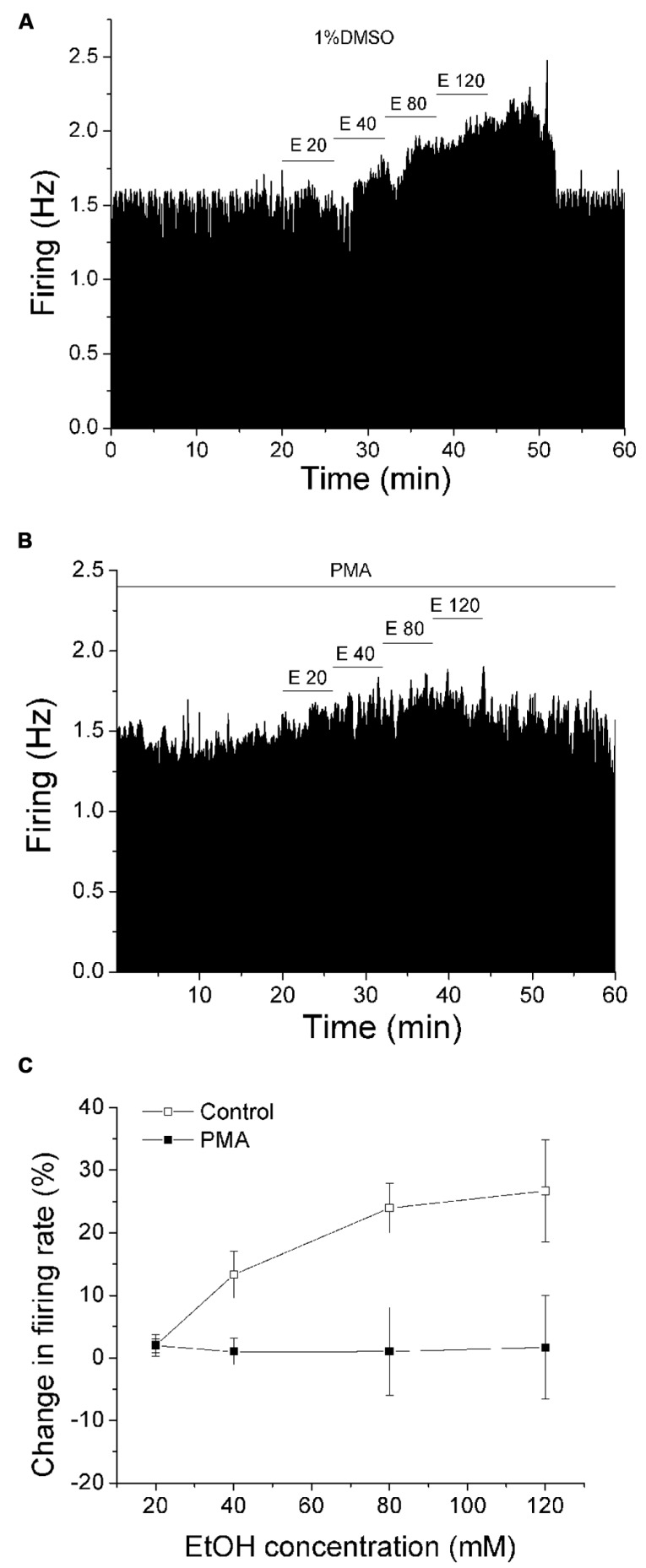
**PMA suppresses ethanol excitation.** **(A,B)** Mean ratemeter graphs of the effects of ethanol application under different conditions on two different DAergic VTA neurons. Vertical bars indicate the firing rate over 5 s intervals. Horizontal bars indicate the duration of drug application (concentrations indicated above bar). Four doses of ethanol (20–120 mM) were applied in a stepwise fashion, in which each concentration was applied for 6 min. **(A)** With 1%DMSO in the recording pipette (control), ethanol produced an increase in firing rate in a concentration-dependent manner. **(B)** With PMA (1 μM) in the recording pipette, there was a blunting of ethanol-induced excitation. **(C)** Pooled results for pDA VTA neurons tested with ethanol in the absence or presence of PMA. With 1% DMSO in the pipette (Control, □), there was a significant concentration-dependent increase in firing rate produced by ethanol (one-way repeated measures ANOVA, *F*_(3,18)_ = 9.99, *p* < 0.05). With PMA in the pipette (PMA, ▪), no significant increase in firing rate produced by ethanol was observed (one-way repeated measures ANOVA, *F*_(3,48)_ = 3.75, *p* > 0.05).

We have previously reported a phenomenon of DIR during extended periods of exposure to moderate concentrations of dopamine, which requires the concurrent stimulation of D2 and D1/D5 dopamine receptors through a conventional PKC pathway (Nimitvilai and Brodie, 2010; Nimitvilai et al., 2012a), and this phenomenon is suppressed by exogenous ethanol (Nimitvilai et al., 2012b). As the primary effect of ethanol on DAergic VTA neurons is to increase the firing rate (Brodie et al., 1990), in the present study, we assessed whether the effect of ethanol was altered by activation of PKC with PMA. Recordings were made with normal saline containing either 1%DMSO (control) or 1 μM PMA in the recording pipette. The firing rate was measured for at least 20 min to allow drug to act locally to the DAergic neuron. Then ethanol was administered in a step-wise fashion from 20 to 120 mM, in which each concentration was applied for 6 min. **Figure 1A** illustrates an experiment in which 1% DMSO is included in the pipette, and the response of a typical DAergic VTA neuron exposed to increasing ethanol concentrations (20–120 mM). Ethanol produced an increase in firing rate in a concentration-dependent manner in this neuron. **Figure 1B** illustrates the effect of PMA (1 μM) on ethanol excitation. In this case, the excitatory effect of ethanol was suppressed at all concentrations. The pooled data of experiments similar to these examples are summarized in **Figure 1C**. Under control conditions with 1% DMSO included in the recording pipette (□, *n* = 7), ethanol at 20, 40, 80, and 120 mM produced a significant increase in firing rate of 2.0 ± 1.74%, 13.29 ± 3.74%, 23.94 ± 3.95%, and 26.68 ± 8.18%, respectively; (one-way repeated measures ANOVA, *F*_(__3,18__)_ = 9.99, *p* < 0.05). With 1 μM PMA in the recording pipette (▪, *n* = 17), no significant excitation was produced by ethanol; ethanol at 20, 40, 80, and 120 mM caused a change in firing rate of 1.92 ± 1.07%, 0.97 ± 2.1%, 1.01 ± 7.07%, and 1.67 ± 8.26%, respectively (one-way repeated measures ANOVA, *F*_(3,48)_ = 3.75, *p* > 0.05).

### PMA INHIBITION OF ETHANOL-INDUCED EXCITATION IS PREVENTED BY A BROAD-SPECTRUM, BUT NOT CONVENTIONAL, PKC INHIBITOR

**FIGURE 2:**
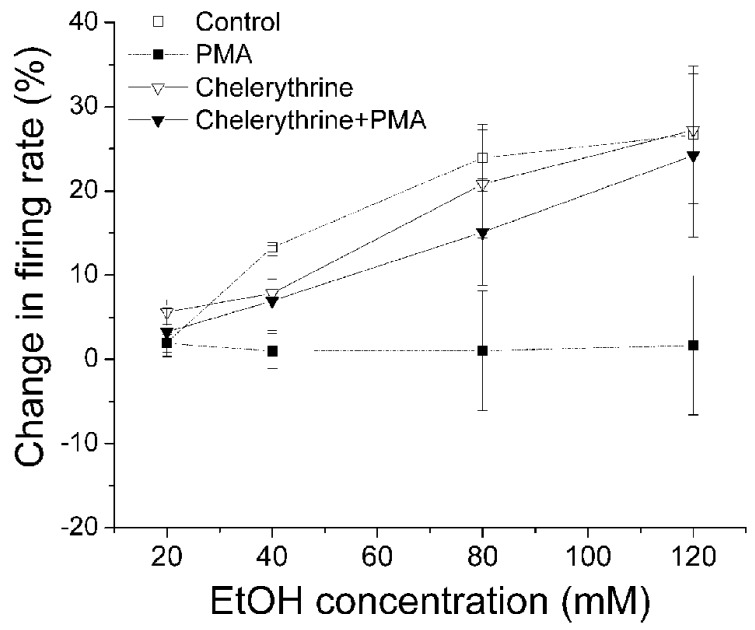
**Chelerythrine inhibits PMA suppression of ethanol-induced excitation.** Percentage change in firing rate (mean ± SEM) in response to ethanol application under different conditions is plotted as a function of time. The effect of ethanol alone (□), and ethanol in the presence of PMA (▪) from **Figure 1C** are shown for comparison. With chelerythrine (10 μM) in the bath and PMA (1 μM) in the recording pipette, there was a significant increase in firing rate produced by increased concentrations of ethanol (▼, *n* = 15; one-way repeated measures ANOVA, *F*_(3,42)_ = 5.64, *p* < 0.05). With chelerythrine (10 μM) in the bath and 1% DMSO in the pipette, ethanol produced a significant concentration-dependent increase in firing rate (▽, *n* = 7; one-way repeated measures ANOVA, *F*_(3,18)_ = 7.69, *p* < 0.05).

Since PMA is a diacylglycerol analog and is an activator of conventional and novel PKCs, we examined the effects of PKC inhibitors on the PMA-induced suppression of ethanol excitation to determine which PKC isoform is required to reduce ethanol excitation. A broad spectrum PKC inhibitor chelerythrine (10 μM) was applied in the bath, with the inclusion of either PMA (1 μM) or 1%DMSO in the recording pipette (**Figure 2**). After 20 min exposure to PMA or DMSO via the pipette, concentrations of ethanol (20–120 mM) were applied. In the presence of chelerythrine, PMA failed to inhibit ethanol excitation; ethanol produced a significant increase in firing rate (▼, *n* = 15; one-way repeated measures ANOVA, *F*_(3,42)_ = 5.64, *p* < 0.05). Chelerythrine alone had no effect on ethanol-induced increase in firing rate (▽, *n* = 7; one-way repeated measures ANOVA, *F*_(3,18)_ = 7.69, *p* < 0.05).

**FIGURE 3:**
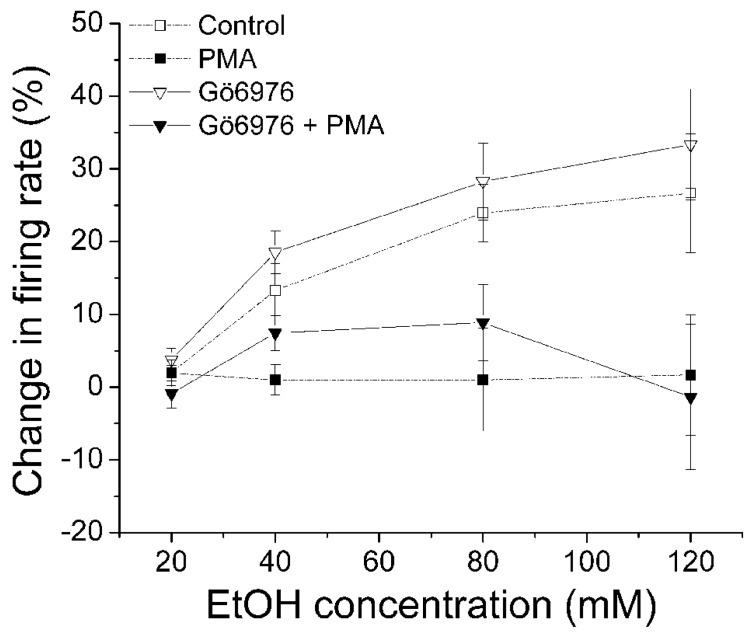
**Gö6976 does not inhibit PMA suppression of ethanol-induced excitation.** Percentage change in firing rate (mean ± SEM) in response to ethanol application in different conditions is plotted as a function of time. The effect of ethanol alone (□), and ethanol in the presence of PMA (▪) from **Figure 1C** are shown for comparison. In the presence of Gö6976 (10 μM) and PMA (1 μM) in the recording pipette, ethanol did not cause a significant increase in firing rate at all concentrations (▼, *n* = 5; one-way repeated measures ANOVA, *F*_(3,21)_ = 0.92, *p* > 0.05). With Gö6976 (10 μM) in the recording pipette, a significant increase in firing rate produced by increased concentrations of ethanol was observed (▽, *n* = 11; one-way repeated measures ANOVA, *F*_(3,12)_ = 8.03, *p* < 0.05).

Then we examined whether a selective inhibitor of conventional PKC, Gö6976, suppressed PMA inhibition of ethanol-induced excitation of DAergic VTA neurons. Gö6976 (10 μM) was co-applied with PMA (1 μM) in the recording pipette (**Figure 3**). We have shown that DIR is blocked by 1 μM Gö6976 in bath (Nimitvilai et al., 2012a) and by 10 μM Gö6976 in the pipette (Nimitvilai et al., 2012c). Therefore, the effect of 10 μM Gö6976 in the pipette is approximately equivalent to the effect of 1 μM Gö6976 applied in the bath solution (see Materials and Methods), and at that concentration, it blocks the activity of conventional PKCs, but not novel or atypical PKCs (Martiny-Baron et al., 1993). In the presence of Gö6976 and PMA, ethanol did not cause a significant increase in firing rate at any concentrations (▼, *n* = 5; one-way repeated measures ANOVA, *F*_(3,21)_ = 0.92, *p* > 0.05), suggesting a lack of involvement effect of conventional PKCs. Ethanol did produce a dose-dependent increase in firing rate when Gö6976 alone was included in the pipette (▽, *n* = 11; one-way repeated measures ANOVA, *F*_(3,12)_ = 8.03, *p* < 0.05).

### PMA INHIBITION OF ETHANOL-INDUCED EXCITATION IS SUPPRESSED BY A SPECIFIC INHIBITOR OF PKCδ/θ

**FIGURE 4:**
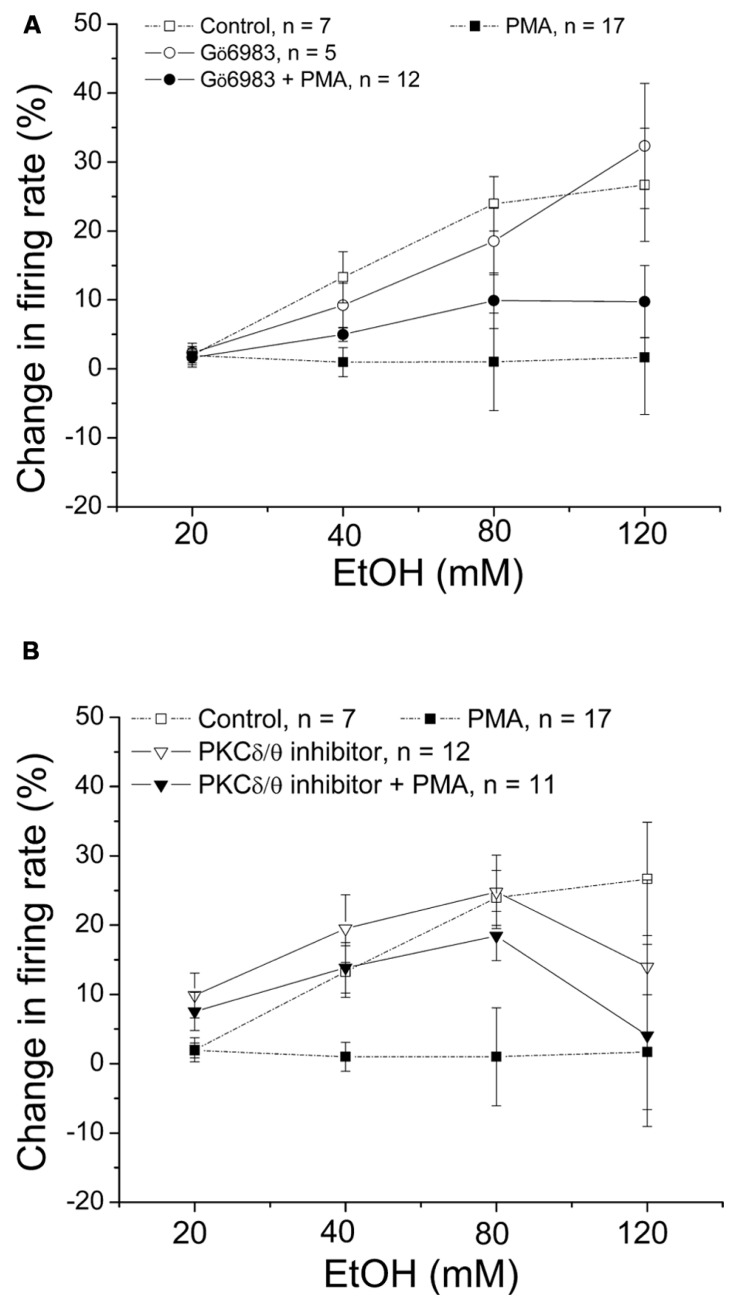
**PKCδ/θ inhibitor, but not Gö6983, inhibits PMA suppression of ethanol excitation.** Percentage change in firing rate (mean ± SEM) in response to ethanol application in different conditions is plotted as a function of time. The effect of ethanol alone (□), and ethanol in the presence of PMA (▪) from **Figure 1C** are shown for comparison. **(A)** With Gö6983 (10 μM) and PMA (1 μM) in the recording pipette, ethanol did not cause a significant increase in firing rate at any concentration (•, *n* = 12; one-way repeated measures ANOVA, *F*_(3,33)_ = 2.26, *p* > 0.05). With Gö6983 (10 μM) alone in the recording pipette, a significant increase in firing rate produced by increased concentrations of ethanol was observed (o, *n* = 5; one-way repeated measures ANOVA, *F*_(3,12)_ = 10.97, *p* < 0.05). **(B)** With PKCδ/θ inhibitor (700 nM) and PMA (1 μM) in the recording pipette, increasing concentrations of ethanol caused significant excitation (▼, *n* = 8; one-way repeated measures ANOVA, *F*_(3,21)_ = 3.78, *p* < 0.05). Similarly, a significant, concentration-dependent increase in firing rate was produced ethanol with PKCδ/θ inhibitor (700 nM) alone in the recording pipette (▽, *n* = 11; one-way repeated measures ANOVA, *F*_(3,30)_ = 19.36, *p* < 0.05).

Gö6983 is a broad spectrum PKC inhibitor that has affinity for conventional PKCs (IC_50_ = 7 nM) and PKCδ (IC_50_ = 10 nM) 300–2,000 times greater than its affinity for other PKCs (Gschwendt et al., 1996). In this experiment, we examined whether Gö6983 blocks the effect of PMA on ethanol excitation. Gö6983 (10 μM) was co-applied with either PMA (1 μM) or 1%DMSO in the recording pipette, and the firing rate was measured 20 min before the addition of ethanol (20–120 mM; **Figure 4A**). In the presence of Gö6983 and PMA, ethanol produced no significant increase in firing rate (•, *n* = 12; one-way repeated measures ANOVA, *F*_(3,33)_ = 2.26, *p* > 0.05). Similarly, Gö6983 alone did not interfere with the excitatory effect of ethanol; ethanol produced a concentration-dependent increase in firing rate (o, *n* = 5; one-way repeated measures ANOVA, *F*_(3,12)_ = 10.97, *p* < 0.05). These results suggest that conventional PKCs and PKCδ do not participate in the mechanism of PMA inhibition of ethanol-induced excitation.

Then we tested whether a PKCδ/θ inhibitor would block the PMA-induced reduction of ethanol excitation (**Figure 4B**). PKCδ/θ inhibitor is a potent inhibitor of PKCθ (IC_50_ = 70 nM) over other novel PKCs (IC_50_ = 0.35, 2.33, and 16.35 μM against δ, ε, and η isoforms, respectively), as well as conventional- and atypical- PKCs (IC_50_ > 50 μM; Cole et al., 2008). In the presence of PKCδ/θ inhibitor (700 nM) and PMA (1 μM) in the recording pipette, PMA failed to suppress the excitatory effect of ethanol; there was a significant increase in firing rate produced by ethanol (▼, *n* = 8; one-way repeated measures ANOVA, *F*_(3,21)_ = 3.78, *p* < 0.05). Likewise, when PKCδ/θ inhibitor (700 nM) was co-applied with 1%DMSO in the pipette, ethanol produced an increase in firing rate of DAergic VTA neurons (▽, *n* = 11; one-way repeated measures ANOVA, *F*_(3,30)_ = 19.36, *p* < 0.05). Although it was a consistent effect, we have no explanation for the sudden decrease in ethanol-induced excitation of firing rate in both conditions when the highest dose (120 mM) of ethanol was applied. Since the low concentration of PKCδ/θ inhibitor (700 nM in the recording pipette, equivalent to 70 nM in the bath) is selective for PKCθ, this result suggests the involvement of PKCθ on PMA reversal of ethanol excitation.

### PMA INHIBITION OF ETHANOL-INDUCED EXCITATION WAS NOT ALTERED BY SUPPRESSION OF CALCIUM RELEASE

**FIGURE 5:**
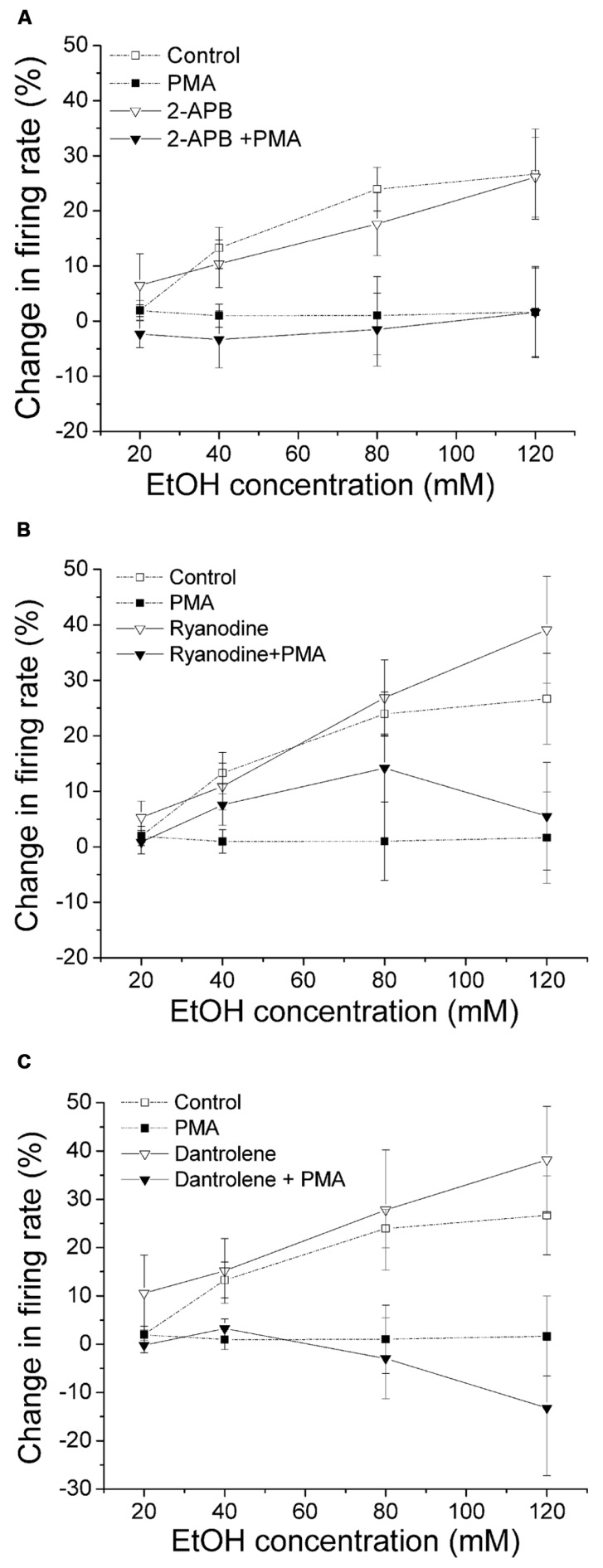
**Inhibition of calcium release from the intracellular stores does not alter PMA suppression of ethanol-induced excitation.** Percentage change in firing rate (mean ± SEM) in response to ethanol application in different conditions is plotted as a function of time. The effect of ethanol alone (□), and ethanol in the presence of PMA (▪) from **Figure 1C** are shown for comparison. **(A)** In the presence of IP3 receptor inhibitor 2-APB (10 μM) in the bath and PMA (1 μM) in the recording pipette, ethanol did not produce a significant increase in firing rate (▼, *n* = 8; one-way repeated measures ANOVA, *F*_(3,21)_ = 0.34, *p* > 0.05). The presence of 2-APB alone in the bath did not alter the excitatory effect of ethanol; there was a significant increase in firing rate produced by ethanol at all concentrations (▽, *n* = 10; one-way repeated measures ANOVA, *F*_(3,27)_ = 19.87, *p* < 0.05). **(B)** With ryanodine (10 μM) in the bath and PMA (1 μM) included in the recording pipette, 80 mM ethanol produced a significant increase in firing rate (one-way repeated measures ANOVA, *F*_(3,21)_ = 2.92, *p* < 0.05), but other doses of ethanol did not significantly change the cell firing (▼, *n* = 8). In the presence of ryanodine alone, ethanol caused a significant increase in firing rate in a concentration-dependent manner (▽, *n* = 7; one-way repeated measures ANOVA, *F*_(3,18)_ = 14.56, *p* < 0.05). **(C)** When dantrolene (10 μM) was applied in the bath with PMA (1 μM) in the recording pipette, ethanol did not produce significant excitation (▼, *n* = 10; one-way repeated measures ANOVA, *F*_(3,27)_ = 0.34, *p* > 0.05). When dantrolene alone was present, ethanol produced a significant concentration-dependent increase in firing rate (▽, *n* = 5; one-way repeated measures ANOVA, *F*_(3,12)_ = 17.07, *p* < 0.05).

Unlike conventional PKC (Nimitvilai et al., 2012a), the activity of PKCθ is not dependent on calcium, therefore interfering with calcium should not affect the PMA inhibition of ethanol-induced excitation. In this experiment, we used agents that inhibit calcium release from the intracellular store and examine whether there was a change in PMA effect on ethanol excitation. In **Figure 5A**, 2-APB was used to block IP3 receptor. With 2-APB alone in the bath, ethanol induced a significant increase in firing rate (▽, *n* = 10; one-way repeated measures ANOVA, *F*_(3,27)_ = 19.87, *p* < 0.05). In the presence of 2-APB (10 μM) in the bath and PMA (1 μM) in the recording pipette, no significant change of firing rate in response to an increased concentrations of ethanol was observed (▼, *n* = 8; one-way repeated measures ANOVA, *F*_(3,21)_ = 0.34, *p* > 0.05). In **Figure 5B**, we tested whether blocking ryanodine receptor by ryanodine produced a change in PMA suppression of ethanol excitation. With ryanodine (10 μM) in the bath and PMA (1 μM) in the recording pipette, 80 mM ethanol produced a small but significant increase in firing rate (one-way repeated measures ANOVA, *F*_(3,21)_ = 2.92, *p* < 0.05), while other doses of ethanol did not cause excitation (▼, *n* = 8). A significant dose response increase in ethanol excitation was observed when only ryanodine was present (▽, *n* = 7; one-way repeated measures ANOVA, *F*_(3,18)_ = 14.56, *p* < 0.05). Because the PMA effect in the presence of ryanodine was not as robust as with PMA alone, we used another ryanodine receptor antagonist, dantrolene, to examine its effect on PMA reversal of ethanol excitation. As shown in **Figure 5C**, in the presence of dantrolene (10 μM) in the bath and PMA (1 μM) in the recording pipette, ethanol did not produce an increase in firing rate (▼, *n* = 10; one-way repeated measures ANOVA, *F*_(3,27)_ = 0.34, *p* > 0.05). Without PMA, in the presence of dantrolene, ethanol produced a significant increase in firing rate in a concentration-dependent manner (▽, *n* = 5; one-way repeated measures ANOVA, *F*_(3,12)_ = 17.07, *p* < 0.05). In contrast to ryanodine, dantrolene did not restore ethanol excitation in the presence of PMA at any concentration of ethanol, possibly due to the fact that ryanodine blocks the ryanodine receptor in a partially open state (Sutko et al., 1997).

The results with dantrolene and 2-APB suggest that the effect of PMA suppression of ethanol-induced excitation is not dependent on intracellular calcium stores. These results also support an involvement of novel, but not conventional, PKCs.

### DOPAMINE INHIBITION REVERSAL DID NOT ALTER THE EXCITATORY EFFECT OF ETHANOL

**FIGURE 6:**
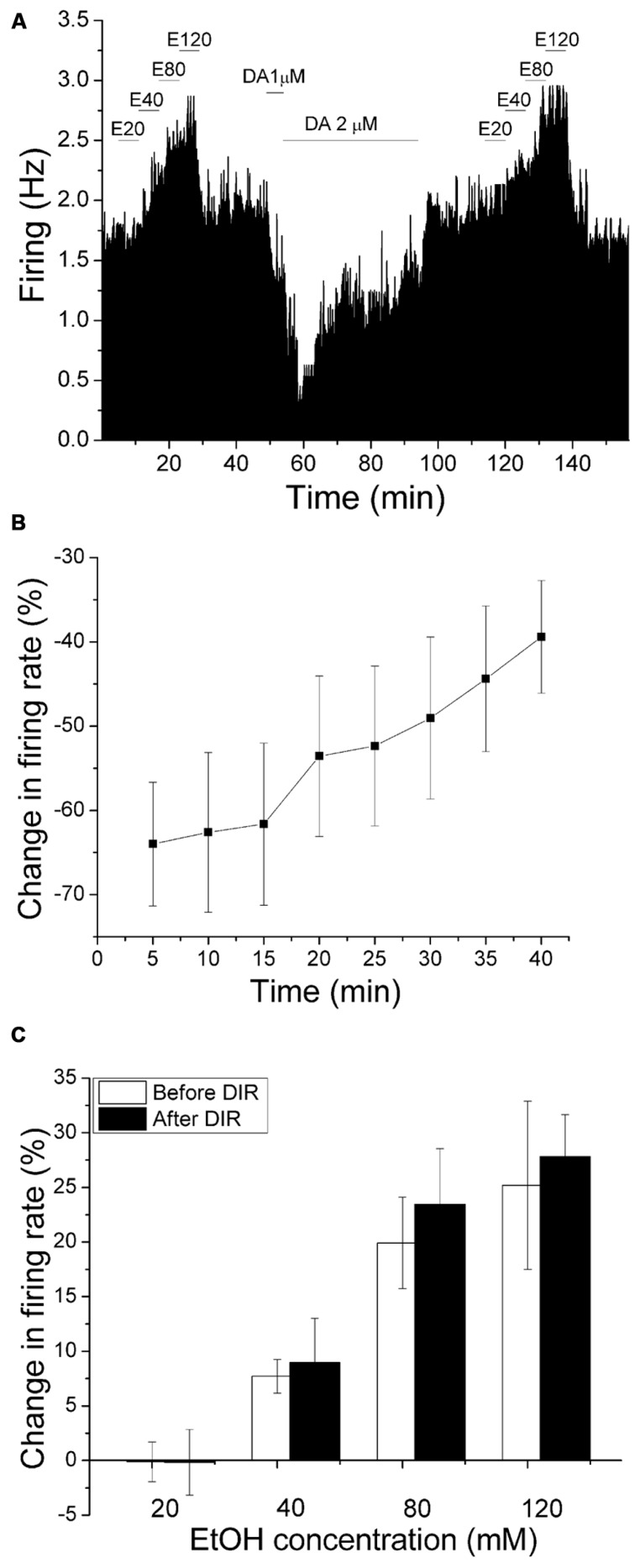
**Dopamine inhibition reversal does not change the excitatory effect of ethanol.** **(A)** Mean ratemeter graph of the effects of ethanol application before and after dopamine inhibition reversal. Vertical bars indicate the firing rate over 5 s intervals. Horizontal bars indicate the duration of drug application (concentrations indicated above bar). Four doses of ethanol (20–120 mM) were applied in a stepwise fashion, in which each concentration was applied for 6 min. After washout of ethanol for 30 min, dopamine (2 μM) was applied for 40 min; dopamine produced an initial inhibition in firing rate with maximum inhibition of 70.55% at 5 min, followed by a partial decrease in dopamine-induced inhibition over the time course. The same concentrations of ethanol were tested again 30 min following the end of the dopamine application. Note that the excitatory effects of increased ethanol concentrations before and after DIR were similar. **(B)** The mean firing rate of DAergic VTA neurons during the 40 min dopamine administration in experiments similar to the one depicted in **(A)**. Concentrations of DA were added in a stepwise fashion until inhibition of 50% or greater was achieved, and these concentrations were applied for 40 min ([DA] = 3.3 ± 0.54 μM, *n* = 5). There was a decrease in the inhibitory effect of dopamine over time; the firing rate at the last three time points were significantly different from the firing rate at the 5 min time point (one-way repeated measures ANOVA, *F*_(7,28)_ = 12.11, *p* < 0.05). **(C)** Comparison of the increase in firing rate produced by ethanol before and after DIR. No significant change in the excitatory effect of ethanol was observed at any concentration (Two-way repeated measures ANOVA, *F*_(1,4)_ = 28.98, *p* > 0.05 for the effect of DIR).

We have shown previously that DIR is dependent on calcium (Nimitvilai et al., 2012a), and is suppressed by exogenous ethanol (Nimitvilai et al., 2012b). As the effect of PMA inhibition of ethanol excitation shown in this study did not require the activation of calcium-dependent PKCs (**Figure 4**), it is likely that calcium-dependent reversal of dopamine inhibition has no effect on ethanol-induced excitation. In the present experiment, we compared the excitatory effect of increased concentrations of ethanol before and after production of DIR. An experiment examining the effect of DIR on ethanol excitation is shown in **Figure 6A**. Concentrations of ethanol were applied in a stepwise fashion (20–120 mM), in which each concentration was applied for 6 min. After washing out ethanol for 30 min, dopamine (2 μM) was administered for 40 min; dopamine produced an initial inhibition in firing rate with maximum inhibition of 70.55% at 5 min, followed by the reversal of dopamine-induced inhibition over time. The same concentrations of ethanol were tested again 30 min following the end of the dopamine application; our previous studies indicate that desensitization of D2 receptors persists for at least 90 min following DIR (Nimitvilai et al., 2010). As shown in **Figure 6A**, there was no reduction in ethanol excitation, compared to the excitatory effect of ethanol before DIR. **Figure 6B** shows the effect of dopamine over time in a pool of experiments similar to the one shown in **Figure 6A**. There was a significant decrease in the inhibitory effect of dopamine at the last three time points compared to the 5 min time point, indicating DIR ([DA] = 3.3 ± 0.54 μM *n* = 5; one-way repeated measures ANOVA, *F*_(7,28)_ = 12.11, *p* < 0.05). **Figure 6C** shows the mean response of ethanol in this paradigm. Before DIR, ethanol at 20, 40, 80, and 120 mM produced an increase in firing rate of -0.12 ± 1.82, 7.72 ± 1.54, 19.91 ± 4.18, and 25.19 ± 7.7%, respectively. After DIR, ethanol at 20, 40, 80, and 120 mM produced an increase in firing rate of -0.16 ± 3.0, 8.96 ± 4.06, 23.44 ± 5.08, and 27.84 ± 3.83%, respectively. In both cases, there was a significant excitatory effect of increasing concentrations of ethanol but no effect of DIR (Two-way repeated measures ANOVA, *F*_(1,4)_ = 28.98, *p* > 0.05 for the effect of DIR). This result suggests that activation of the calcium-dependent PKC required for the induction of DIR did not interfere with ethanol excitation, and so the PKC isoform involved in DIR is different from that involved in reduction of ethanol excitation.

## DISCUSSION

In the results presented above, we report that PMA inhibits the excitatory effect of increasing concentrations (20–120 mM) of ethanol through a calcium-independent novel PKC mechanism; the PKC isoform PKCθ is likely to mediate this reduction of ethanol activation of DA VTA neurons. The present report emerged from our observation that dopamine D2 receptor desensitization, termed DIR, is blocked by low concentrations of ethanol (Nimitvilai et al., 2012b). In that study, we preliminarily commented that the excitatory effect of ethanol was suppressed by the addition of PMA (Nimitvilai et al., 2012b). As there is a rich literature on the effects of ethanol on PKC, and alteration of neurotransmitter effects by either PKC or ethanol (Stubbs and Slater, 1999; Newton and Messing, 2006), we felt that it was important to examine the effect of PKC activation on ethanol excitation of DAergic VTA neurons.

The primary effect of ethanol on the VTA is to increase the firing rate of the DAergic neurons both *in vivo* (Gessa et al., 1985) and *in vitro* (Brodie et al., 1990). In brain slices, ethanol can produce excitation of VTA neurons in low-calcium and high-magnesium buffer, suggesting a direct excitatory action of ethanol on DAergic cells (Brodie et al., 1990). The observed PMA inhibition of ethanol-induced excitation in this study suggests that activation of either conventional- or novel- PKCs may regulate the ethanol excitation since both conventional- and novel- PKCs are phorbol ester-sensitive, while atypical PKCs are resistant to PMA. However, this inhibitory effect of PMA on EtOH excitation still occurred when the release of calcium from intracellular stores was suppressed or a specific inhibitor of conventional PKCs like Gö6976 (10 μM) was applied, so it is unlikely that conventional PKCs participate in this phenomenon. Gö6976 has a high affinity for conventional PKCs with IC_50_ less than 7 nM (Martiny-Baron et al., 1993) so that the 10 μM in the recording pipette (equivalent to 1 μM in bath) used in this study should be sufficient to inhibit the function of conventional PKCs. Similarly, Gö6983 has a high affinity for conventional PKCs with IC_50_ about 7 nM, and it also shows a high specificity for PKCδ with IC_50_ of 10 nM. The inability of Gö6983 to attenuate the PMA effect on ethanol excitation indicates that PKCδ and conventional PKCs do not participate in this phenomenon. As an additional observation ruling out conventional PKCs, ethanol-induced excitation was not attenuated after development of DIR, which we have shown involves activation of conventional PKCs.

The PKCδ/θ inhibitor (700 nM in the recording pipette) significantly blocked the inhibitory effect of PMA on ethanol excitation. The PKCδ/θ inhibitor has IC_50_ of 70 nM, 0.35 μM, and 2.33 μM against PKC-θ, -δ, and -ε, respectively. As Gö6983 has a high affinity for PKCδ and did not significantly block the PMA suppression of ethanol excitation, mediation of this effect of PMA by PKCδ can be ruled out. Thus, these experiments support the role of PKCθ as the isoform of PKC that mediates the PMA-induced reduction of ethanol excitation of DA VTA neurons. While our conclusion is based on the pharmacological specificity of the agents used, there may be other non-specific actions of these agents that may confound our interpretation. Similarly, the precise concentration of the PKC antagonists to which the cells are exposed is unknown, due to diffusion barriers in the brain slice and from the micropipettes to the cells. Additional studies using techniques such as gene knock-out may be necessary to identify conclusively which PKC isoform is required to inhibit the excitatory effect of ethanol in the DA VTA neurons.

**FIGURE 7:**
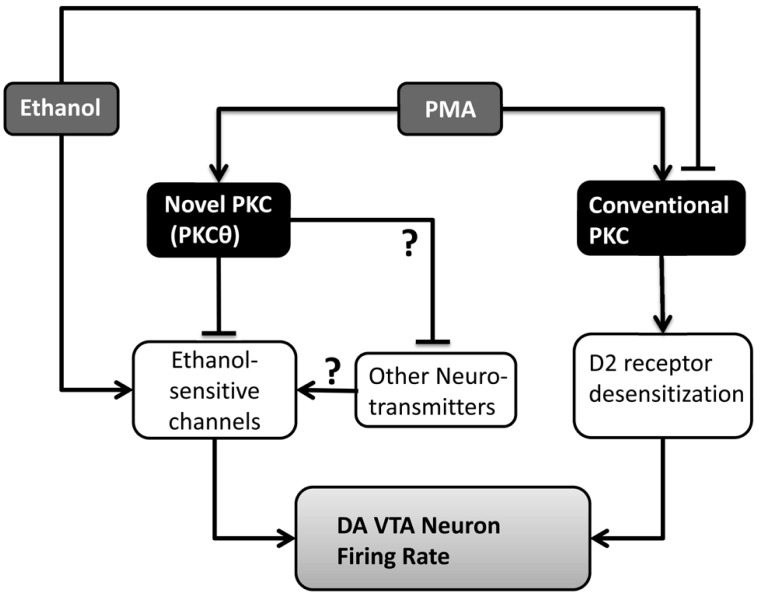
**Model of the interactions of PMA and ethanol in the VTA.** PMA activates two isoforms of PKC: PKCθ and a conventional PKC responsible for D2 receptor desensitization. Ethanol inhibits the conventional PKC, and affects a number of ion channels (ethanol-sensitive channels) increasing the firing rate of DA VTA neurons. PKCθ interferes with the action of ethanol on ion channels in the DA VTA neuronal membrane, preventing the excitation. Unknown is whether other neurotransmitters interact with those same ethanol-sensitive ion channels and whether PKCθ alters those neurotransmitters as well.

A schematic model for the interactions of PMA and ethanol on DA VTA neurons is shown in **Figure 7**. In the presence of PMA before the addition of ethanol, both a conventional PKC (responsible for DIR) and PKCθ are activated. Activation of that conventional PKC in the presence of dopamine increases desensitization of D2 receptors, causing an overall increase in activity or excitability. Activation of PKCθ does not have a significant effect on DA VTA neuronal activity by itself. Ethanol in the absence of PMA has two effects in this model: inhibition of the conventional PKC to prevent D2 desensitization, and action on a number of ion channels (as enumerated in the Introduction, e.g., blockade of M-current) to increase firing rate. When PMA and ethanol are present, ethanol reduces the effect of PMA to promote DIR and, at the same time, the effects of ethanol on ion channels to increase firing rate is blocked by PMA-activated PKCθ. Actions of ethanol on other neurotransmitter systems that may involve PKCθ are unknown at this time. The observations that ethanol inhibits a conventional PKC and that activation of PKCθ inhibits ethanol-induced excitation underscore the importance of studying different subtypes of PKC to better understand the complexity of PKC-ethanol interactions in the VTA.

Recent studies indicate that ethanol activation of DAergic VTA neurons is mediated by salsolinol via the synthesis of acetaldehyde from ethanol. Dopamine neuron excitation (Melis et al., 2007; Diana et al., 2008)as well as effects on dopamine release *in vivo* (Deehan et al., 2013), can be produced by acetaldehyde, and the excitatory effects of ethanol are attenuated by the catalase inhibitor 3-AT (Melis et al., 2013). In addition, salsolinol, a tetrahydroisoquinoline that is a condensation produce of dopamine and acetaldehyde, is a substrate for self-administration directly into the VTA (Rodd et al., 2008), increases dopamine release (Hipolito et al., 2009), and increases the firing rate of DAergic VTA neurons (Xie et al., 2013). Salsolinol excites DAergic VTA neurons after depletion of DA, whereas both ethanol and acetaldehyde appear to require endogenous dopamine, further supporting the role of salsolinol as the agent directly mediating ethanol excitation (Melis et al., 2013). Interestingly, salsolinol appears to act both pre- and post-synaptically to increase DAergic neuronal excitability and firing rate (Xie et al., 2013). Extensive studies will have to be performed to determine whether PMA suppresses salsolinol-induced excitation, and to identify the specific pre- or post-synaptic sites involved in any PMA-salsolinol interaction.

It is possible that PKCθ reduces ethanol excitation by altering an ionic conductance or neurotransmitter effect that masks the excitatory action of ethanol. There is precedent for concurrent processes that could interfere with ethanol-induced excitation of DA VTA neurons: block of h-channels with ZD7288 uncovers ethanol-induced activation of barium-sensitive potassium channels that undermines ethanol excitation of DA VTA neurons (Okamoto et al., 2006; McDaid et al., 2008). It is possible that PKCθ opens or closes channels that shunt the excitatory ethanol current, or alter the membrane resistance to reveal an inhibitory effect of ethanol that masks the excitation. Investigations into the cellular localization of PKCθ and the ion channels altered by PKCθ will be needed to understand more regarding the interactions of ethanol and PKCθ in the VTA.

The action of ethanol on PKC has been reported to regulate the functions of numerous receptors and cell activities (Stubbs and Slater, 1999; for review see Newton and Messing, 2006). Ethanol activation of PKC potentiates GABA_A_ responses (Wafford and Whiting, 1992; Weiner et al., 1994; Harris et al., 1995), inhibits AMPA/kainate receptors (Dildy-Mayfield and Harris, 1995), induces tolerance of adenosine A2 receptors (Coe et al., 1996), inhibits or stimulates glycine currents (Ye et al., 2001; Tao and Ye, 2002; Jiang and Ye, 2003), as well as suppresses the functions of 5HT1c and M1 muscarinic receptors (Sanna et al., 1994). In addition, ethanol inhibition of PKC can prevent augmentation of NMDA response (Reneau et al., 2011), and reduce D1 dopamine receptor phosphorylation (Rex et al., 2008). In the present study, we demonstrate an action of PKC activation on ethanol excitation. While ethanol excites DA VTA neurons directly without mediation by synaptic inputs (Brodie et al., 1999b), ethanol excitation may be modulated by other neurotransmitters acting on DA VTA neurons or by direct modulation of ethanol-sensitive ion channels by PKC. Whether PMA suppression of ethanol-induced excitation reported in this study is mediated by PKC phosphorylation of a specific receptor or ion channel is a subject for future study.

The results here indicate that PKCθ is the primary candidate for mediating PMA-induced reduction of ethanol excitation. PKCθ distribution within the central nervous system has not been well described until recently. Initial studies suggested that significant levels of PKCθ were not found in rodent brain (Tanaka and Nishizuka, 1994; Naik et al., 2000), but other studies found significant levels of PKCθ in habenula (Minami et al., 2000). A more recent study examined PKCθ localization within the hypothalamus, but also did a comprehensive examination of localization of PKCθ and PKC δ throughout the brain (Irani et al., 2010). While finding no significant levels of PKCδ in the substantia nigra or VTA, that study observed a high concentration of PKCθ-containing fibers within the VTA. This observation suggests that the effects of PKCθ activation on ethanol-induced excitation may be mediated by synaptic inputs to DA VTA neurons. Additional studies will be necessary to carefully examine the neurochemical identity of synapses that are modulated by PKCθ which could indicate a mechanism by which PMA suppresses ethanol-induced excitation. Elucidating this PKCθ mechanism might reveal a new and important target for treatment of alcohol and drug addiction.

## Conflict of Interest Statement

The authors declare that the research was conducted in the absence of any commercial or financial relationships that could be construed as a potential conflict of interest.

## AUTHOR CONTRIBUTIONS

Participated in research design: Nimitvilai, Arora, You, and Brodie. Conducted experiments: Nimitvilai, Arora, You, and McElvain. Performed data analysis: Nimitvilai, Arora, You, McElvain, and Brodie. Wrote or contributed to the writing of the manuscript: Nimitvilai, Arora You, and Brodie

## ABBREVIATIONS

aCSF: artificial cerebrospinal fluid
DA: dopamine
DAergic: dopaminergic
Gö6976: 5,6,7,13-tetrahydro-13-methyl-5-oxo-12H-indolo[2,3-a]pyrrolo[3,4c]carbazole-12-propanenitrile
Gö6983: 3-[1-[3-(Di methylamino)propyl]-5-methoxy-1H-indol-3-yl]-4-(1H-indol-3-yl)-1H-pyrrole-2,5-dione
PKC: protein kinase C
PKCδ/θ inhibitor: (5-(3,4-Dimethoxyphenyl)-4-(1H-indol-5-ylamino)-3-pyridinecarbonitrile)
PMA: phorbol 12-myristate 13-acetate
VTA: ventral tegmental area
